# Supplementary material for: Exploratory Study of Gastrointestinal Redox Biomarkers in the Presymptomatic and Symptomatic Tg2576 Mouse Model of Familial Alzheimer’s Disease: Phenotypic Correlates and Effects of Chronic Oral d-Galactose
Source: ACS Chem Neurosci. 2023 Nov 6;14(22):4013–25. doi: 10.1021/acschemneuro.3c00495 (PMC10655039; doi:10.1021/acschemneuro.3c00495)
Supplement: Supplementary file 1 — cn3c00495_si_001.pdf [file cn3c00495_si_001.pdf]

Supporting Information

An exploratory study of gastrointestinal redox biomarkers in the presymptomatic and symptomatic Tg2576 mouse model of familial Alzheimer's disease – phenotypic correlates and the effects of chronic oral D-galactose

Jan Homolak[1,2, 3, 4], Ana Babic Perhoc[1,2], Ana Knezovic[1,2], Jelena Osmanovic Barilar[1,2], Davor Virag[1,2], Melita Salkovic-Petrisic[1,2]

[1] Department of Pharmacology, University of Zagreb School of Medicine, Zagreb 10000, Croatia

[2] Croatian Institute for Brain Research, University of Zagreb School of Medicine, Zagreb 10000, Croatia

[3] Interfaculty Institute of Microbiology and Infection Medicine, University of Tübingen, Tübingen 72076, Germany

[4] Cluster of Excellence "Controlling Microbes to Fight Infections", University of Tübingen, Tübingen 72076, Germany

Corresponding author:

Jan Homolak, MD, PhD

Department of Pharmacology

University of Zagreb School of Medicine

Salata 11, 10 000 Zagreb

Croatia

0038591 9411 468

homolakjan@gmail.com

jan.homolak@mef.hr

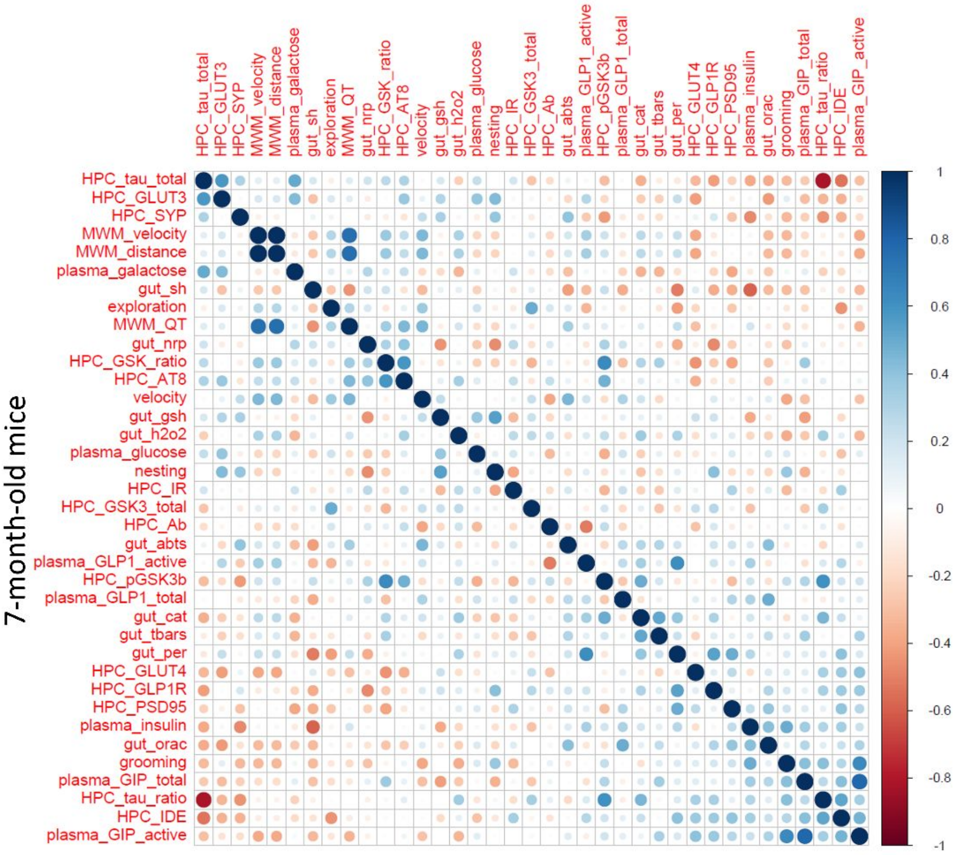
7-month-old mice
HPC_tau_total
HPC_GLUT3
HPC_SYP
MWM_velocity
MWM_distance
plasma_galactose
gut_sh
exploration
MWM_QT
gut_nrp
HPC_GSK_ratio
HPC_AT8
velocity
gut_gsh
gut_h2o2
plasma_glucose
nesting
HPC_IR
HPC_GSK3_total
HPC_Ab
gut_abts
plasma_GLP1_active
HPC_pGSK3b
plasma_GLP1_total
gut_cat
gut_tbars
gut_per
HPC_GLUT4
HPC_GLP1R
HPC_PSD95
plasma_insulin
gut_orac
grooming
plasma_GIP_total
HPC_tau_ratio
HPC_IDE
plasma_GIP_active
1
0.8
0.6
0.4
0.2
0
-0.2
-0.4
-0.6
-0.8
-1

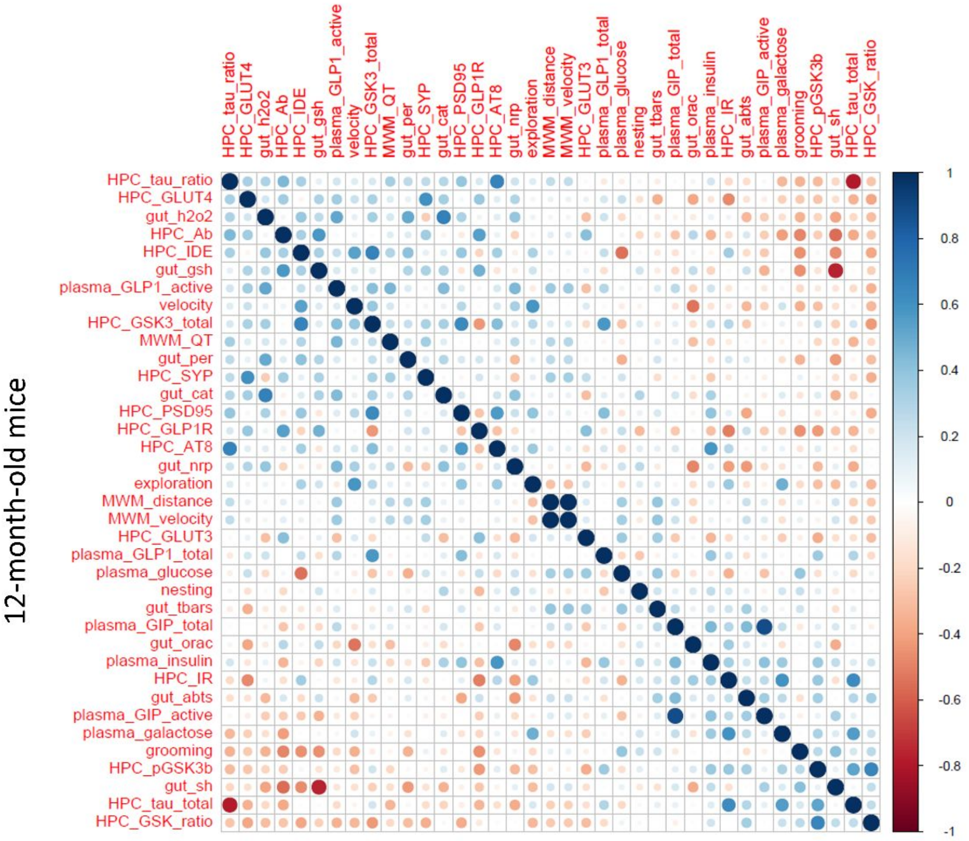
12-month-old mice
HPC_tau_ratio
HPC_GLUT4
gut_h2o2
HPC_Ab
HPC_IDE
gut_gsh
plasma_GLP1_active
velocity
HPC_GSK3_total
MWM_QT
gut_per
HPC_SYP
gut_cat
HPC_PSD95
HPC_GLP1R
HPC_AT8
gut_nrp
exploration
MWM_distance
MWM_velocity
HPC_GLUT3
plasma_GLP1_total
plasma_glucose
nesting
gut_tbars
plasma_GIP_total
gut_orac
plasma_insulin
HPC_IR
gut_abts
plasma_GIP_active
plasma_galactose
grooming
HPC_pGSK3b
gut_sh
HPC_tau_total
HPC_GSK_ratio
1
0.8
0.6
0.4
0.2
0
-0.2
-0.4
-0.6
-0.8
-1

Fig S1. An analysis of correlations between functional and behavioral outcomes, redox biomarkers, and molecules associated with metabolic signaling pathways in the gut and the brains of mice aged 7 and 12 months.
